# Identification and Validation of a Four-Gene Ferroptosis Signature for Predicting Overall Survival of Lung Squamous Cell Carcinoma

**DOI:** 10.3389/fonc.2022.933925

**Published:** 2022-07-07

**Authors:** Qi Wang, Yaokun Chen, Wen Gao, Hui Feng, Biyuan Zhang, Haiji Wang, Haijun Lu, Ye Tan, Yinying Dong, Mingjin Xu

**Affiliations:** ^1^ Department of Radiation Oncology, The Affiliated Hospital of Qingdao University, Qingdao, China; ^2^ Breast Disease Diagnosis and Treatment Center, Qingdao Center Medical Group, Qingdao, China

**Keywords:** lung squamous cell carcinoma, ferroptosis, prognostic model, microenvironment, nomogram

## Abstract

**Background:**

Lung squamous cell carcinoma (LUSC) represents 30% of all non-small cell lung carcinoma. Targeted therapy is not sufficient for LUSC patients because of the low frequency of targeted-effective mutation in LUSC whereas immunotherapy offers more options for patients with LUSC. We explored a ferroptosis-related prognostic signature that can potentially assess the prognosis and immunotherapy efficacy of LUSC patients.

**Methods:**

A total of 502 LUSC patients were downloaded from The Cancer Genome Atlas (TCGA). The external validation data were obtained from the Gene Expression Omnibus (GEO): GSE73403. Then, we identified the candidate genes and constructed the prognostic signature through the Cox survival regression analyses and least absolute shrinkage and selection operator (LASSO). Risk score plot, Kaplan–Meier curve, and ROC curve were used to assess the prognostic power and performance of the model. The CIBERSORT algorithm estimated the fraction of immune cell types. TIDE was utilized to predict the response to immunotherapy. IMvigor210 was used to explore the association between the risk scores and immunotherapy outcomes. A nomogram combined selected clinical characteristics, and the risk scores were constructed.

**Results:**

We screened 132 differentially expressed ferroptosis-related genes. According to KEGG and GO pathway analyses, these genes were mainly engaged in the positive regulation of cytokine production, cytokine metabolic process, and oxidoreductase activity. We then constructed a prognostic model *via* LASSO regression. The proportions of CD8^+^ T cells, CD4^+^ activated T cells, and follicular helper T cells were significantly different between low-risk and high-risk groups. TIDE algorithm indicated that low-risk LUSC patients might profit more from immune checkpoint inhibitors. The predictive value of the ferroptosis gene model in immunotherapy response was further confirmed in IMvigor210. Finally, we combined the clinical characteristics with a LASSO regression model to construct a nomogram that could be easily applied in clinical practice.

**Conclusion:**

We identified a prognostic model that provides an accurate and objective basis for guiding individualized treatment decisions for LUSC.

## Introduction

Lung cancer has the highest morbidity and mortality of all cancers globally (1), and non-small cell lung cancer (NSCLC) comprises approximately 85%. Currently, most lung cancer patients arrived at their first diagnosis with advanced-stage disease, generally because of a lack of typical clinical symptoms. Over the past decade, specific targeted therapy has emerged as the most promising treatment. However, molecular aberrations in specific genes, such as epidermal growth factor receptors, are required for targeted therapies to be effective in lung adenocarcinoma patients (2, 3).

To date, there are no approved targeted therapies for lung squamous cell carcinoma (LUSC). The targeted therapy is not sufficient for LUSC because of the low frequency of targeted-effective mutation in LUSC, whereas immunotherapy offers more options for patients with LUSC. The dramatic development of immune checkpoint inhibitors (ICIs) has marked a revolution in the treatment of LUSC. Early-stage clinical trials have shown that objective response rates to ICIs are approximately 14%–20%. FDA approves pembrolizumab monotherapy as first-line treatment for LUSC patients who has high PD-L1 expression (4, 5). Furthermore, ICIs and chemotherapy have been approved as the first-line treatment (6). However, because of the high tumor heterogeneity of LUSC, the immunotherapy efficacy may differ greatly across LUSC patients (7). Meanwhile, the high price and limited availability of ICIs severely inhibit their clinical use. Thus, the development of new treatment approaches and the exploration of effective prognostic models for screening high-risk patients with LUSC has attracted increasing attention for the past few years.

Ferroptosis is a type of oxidative cell death presented in neurological disorders, blood diseases, and tumors (8). Over the years, it has been proposed that ferroptosis might prove useful as a promising target for killing cancer cells that are resistant to conventional treatment. Many genes validated to be associated with ferroptosis are involved in the regulation of tumorigenesis, such as TP53 (9), SLC7A11 (10), FBXW7 (11), and CISD2 (12). There is an increasing body of literature that recognizes tumor immunity depending on the tumor microenvironment that regulated iron metabolism and hemostasis *in vivo* (13, 14). Zhang (15) found that oxygen radicals induced lethal ferroptosis of tumor, and the resulting tumor antigens enhanced the immunogenicity. Therefore, immunomodulation and ferroptosis performed synergistically to achieve potent therapeutic effects. Thus, the in-depth exploration of biomarkers associated with tumor immunity and ferroptosis could contribute to a comprehensive picture of cancer immunotherapy.

Currently, several studies have established ferroptosis prognostic signatures of cancer from public databases. Lu (16) discovered a novel risk model composed of ferroptosis gene variants in order to predict esophageal squamous cell carcinoma outcomes. Ye (17) built ferroptosis-related genes (FRGs) that were identified to predict prognoses of ovarian cancer and could potentially be used to target new treatments. However, there has been little discussion about the role of FRGs in LUSC patients.

We used the Gene Expression Omnibus (GEO) and The Cancer Genome Atlas (TCGA) databases to construct ferroptosis-related prognostic signatures for LUSC patients. Comparing tumor tissues with adjacent normal tissues in LUSC patients, we determined differentially expressed FRGs. Furthermore, we used the training set to identify survival-related signatures and build an FRG prognostic model. The model’s accuracy and reliability were validated by an external GEO cohort. Overall, researchers examined a ferroptosis-related risk model that could be used to promote better clinical strategies for LUSC patients.

## Methods and Materials

### Data Source

The transcriptomic data of 502 LUSC patients, as well as the corresponding clinical parameters, were downloaded from the TCGA. The external validation data were obtained from GEO: GSE73403 (18). IMvigor210 (19) was used to explore the relation between the risk model and immunotherapy response. The transcriptomic data in each database were normalized to fragments per kilobase of transcript per million mapped reads (FPKMs) and subjected to further log2 transformation using the limma Bioconductor package (20). There were 587 FRGs downloaded from the FerrDb (shown in **Supplementary Table 1**) as candidate genes. The transcriptomic and corresponding clinical factors of IMvigor210 were downloaded *via* “IMvigor210CoreBiologies” R packages (21).

### Identification of Differentially Expressed FRGs

The limma Bioconductor package was used to detect the differentially expressed genes (DEGs) between tumor and normal tissues in the TCGA dataset. The threshold values were set up as follows: log2-fold change ≥ 1 and *p*-value <0.05. Pheatmap, a package for generating heatmaps and volcano plots, was used to generate the heatmap and volcano plot. We used Kyoto Encyclopedia of Genes and Genomes (KEGG) and Gene Ontology (GO) enrichment analyses to examine the possible functions of DEGs (22, 23), which provide a complete set of functional annotation methods for understanding the biological significance of a large numbers of genes. To screen out FRGs, we merged all data by taking the interaction of the DEGs and candidate genes.

### Construction and Validation of the Ferroptosis-Related Gene Model

Univariate Cox regression analysis was conducted in the TCGA cohort to find FRGs that were linked to overall survival (OS) by employing the “survival” R package. Then, the TCGA cohort was randomly divided into the training and testing cohorts at a 7:3 ratio by the “caret” R package. Through the use of the “glmnet” R package (24), we developed a prognostic signature using the least absolute shrinkage and selection operator (LASSO) in the training cohort. The risk score of each LUSC patient was calculated as follows:


Risk score=∑incoefficient×expression offerroptosis related genes

The same formula used in the training set was used to determine the risk scores of patients in the testing and external validation cohorts. On the basis of the median risk score, all LUSC patients were divided into low- and high-risk groups. Using Kaplan–Meier plots, we evaluated and compared the OS time between groups. To determine the prognostic model’s sensitivity and specificity, ROC curves were calculated (by employing the “time ROC” R package) (25).

### Identification of the Relationship Between the Risk Score and the Immune Landscape

CIBERSORT identified the abundances of different cell types using RNAseq profiles of bulk samples (26). In summary, employing signatures from 500 marker genes, CIBERSORT can simultaneously count 22 immune cell types at once and estimate their relative amounts. We used CIBERSORT to compare the amount of tumor-infiltrating immune cells in different groups of LUSC patients.

We selected 8 transcripts to be immune-checkpoint-relevant, namely, CD274, CTLA4, HAVCR2, LAG3, PDCD1, PDCD1LG2, TIGIT, and SIGLEC15, and extracted their expression levels. The expression of risk score and selected immune-checkpoint-relevant genes was illustrated in a heatmap by “pheatmap” R package. The TIDE algorithm was used to estimate the likelihood of an immunotherapy response (27).

### Nomogram Development and Validation

Based on the risk score and clinical parameters, we designed a predictive nomogram to guide clinical decision-making. The survival-related clinical variables were screened at a *p*-value of less than 0.05 *via* univariate Cox analysis. An analysis of multivariate survival was next performed to create a nomogram. The nomogram prediction probabilities were plotted against the measured rates using a calibration curve. The nomogram’s clinical value was investigated using decision curve analysis (DCA) (28). The nomogram, calibration curves, and DCAs were all plotted using the R packages “rms” and “rmda.”

### Statistical Analyses

R Studio software (version 1.4.1717) was used to conduct all analyses. To compare samples from normal and tumor, we used Student’s *t*-test. Where appropriate, the *χ* ^2^ test or Fisher’s test was applied to determine the connection between the risk score and clinical parameters. Kaplan–Meier plots of survival times were analyzed using the log-rank test. All *p*-values were two-tailed with a significance level of 0.05.

## Results

### Screening for 12 DEGs Associated With a Poor Prognosis

**Figure 1:**
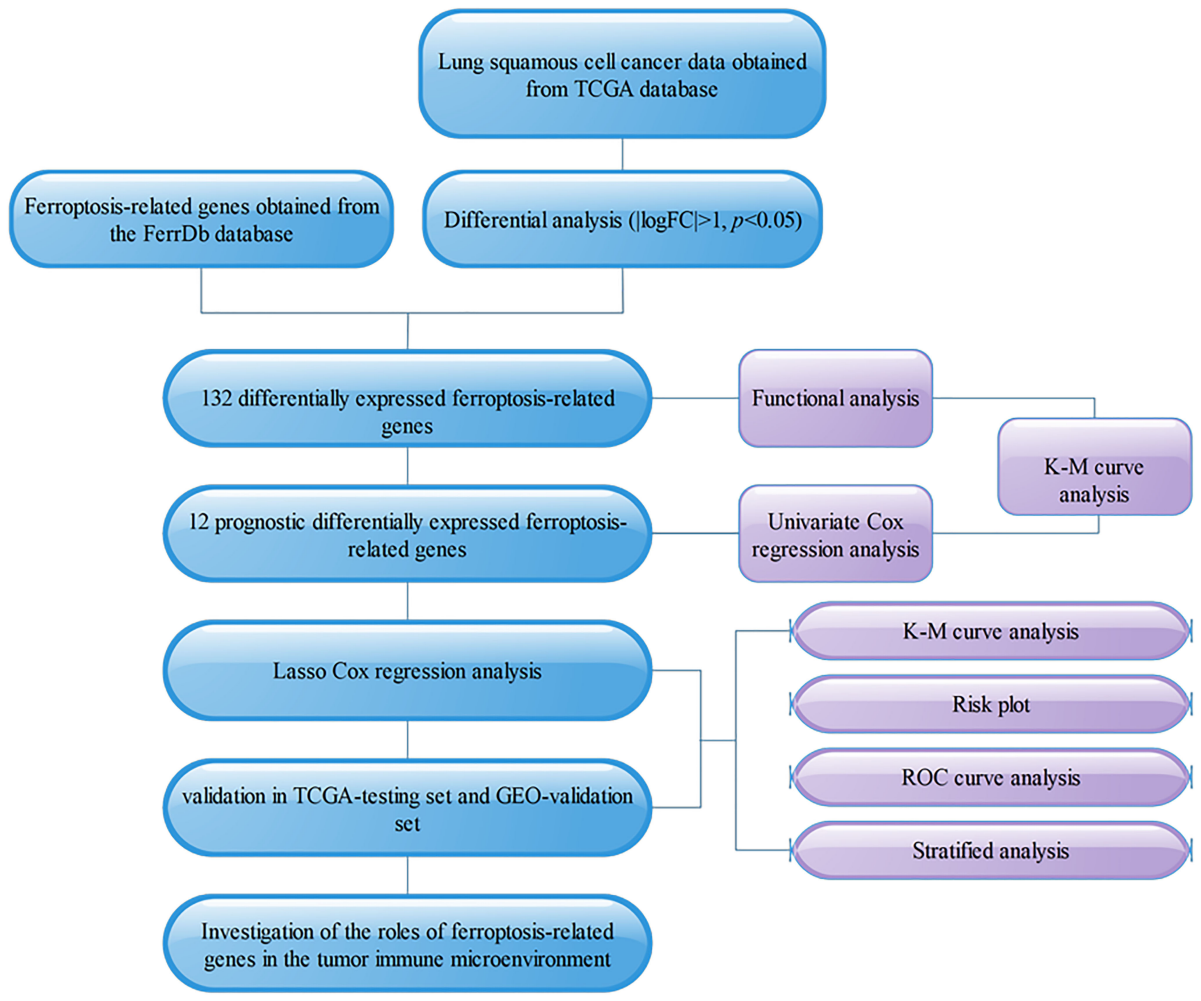
Flowchart.

**Figure 2:**
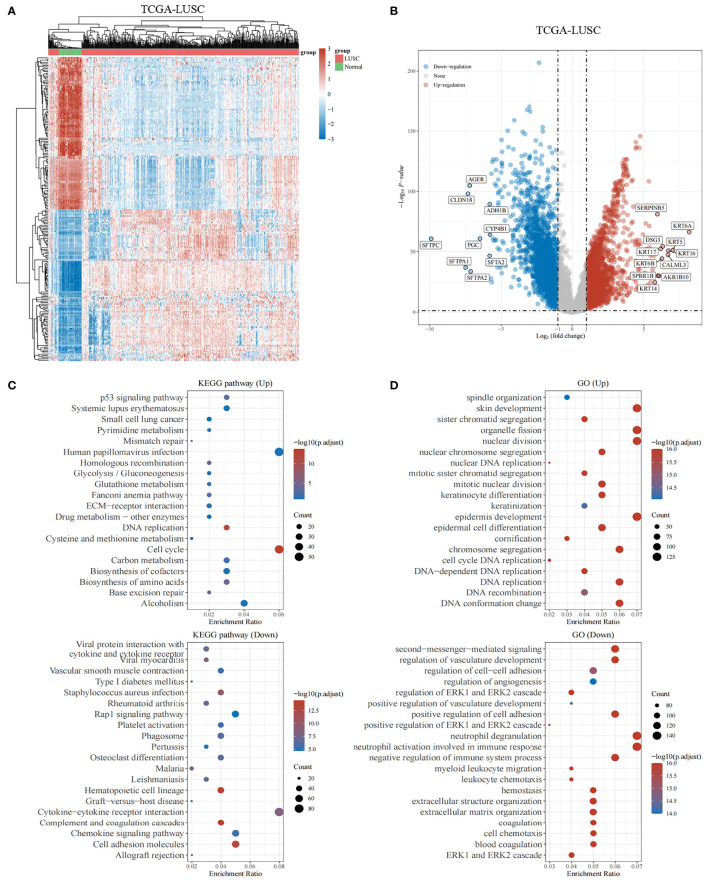
Genes differentially expressed in lung squamous cell carcinoma. **(A, B)** The heatmap and volcano plot shows differentially expressed genes in lung squamous cell carcinomas based on the TCGA database. Up- and downregulated genes are indicated in red and blue, respectively. **(C)** Bubble graph for KEGG pathways. **(D)** Bubble graph for GO pathways.

The study protocol is shown in **Figure 1**. We obtained 16,164 DEGs between LUSC tissues and normal tissues from the TCGA. Among them, 10,623 were downregulated, and 5,541 were upregulated (shown in **Figures 2A, B**). We found that the upregulated DEGs were mainly enriched in systemic lupus erythematosus, small cell lung cancer, pyrimidine metabolism, p53 signaling pathway, mismatch repair, and homologous recombination. The downregulated genes were significantly enriched in pathways including cytokine–cytokine receptor interactions, viral myocarditis, and chemokine signaling pathways (**Figure 2C**). The upregulated DEGs in the GO functional analysis were mainly associated with biological processes involving spindle organization, nuclear division, organelle fission, epidermal cell differentiation, and cell cycle DNA replication. The downregulated DEGs were enriched in regulation of angiogenesis, and regulation of the ERK1 and ERK2 cascades (shown in **Figure 2D**).

**Figure 3:**
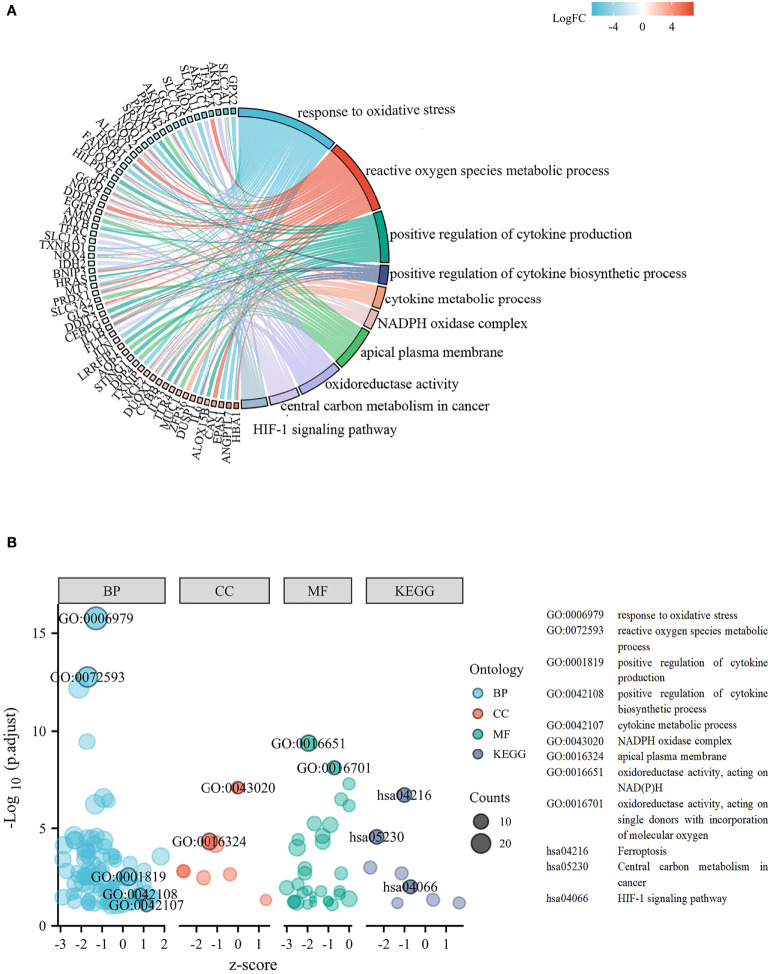
Functional analysis based on the differentially expressed FRGs. **(A)** Chord plot of molecular function and biological process. **(B)** Bubble graph of molecular function and biological process.

The experimentally validated FRGs were crossed with the DEGs. In total, the 132 differentially expressed FRGs were further screened in the TCGA cohort. We performed functional enrichment analysis of differentially expressed FRGs in LUSC. The chord plot and bubble plot showed that the FRGs were mainly enriched in the response to oxidative stress, positive regulation of cytokine production, cytokine metabolic process, oxidoreductase activity, and HIF-1 signaling pathway (shown in **Figures 3A, B**).

**Figure 4:**
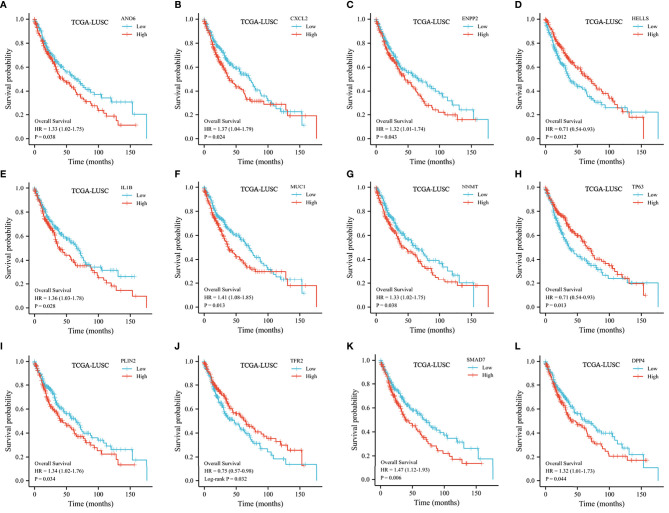
Kaplan–Meier curve of the selected FRGs from the TCGA dataset. **(A)** Kaplan–Meier curve according to ANO6. **(B)** Kaplan–Meier curve according to CXCL2. **(C)** Kaplan–Meier curve according to ENPP2. **(D)** Kaplan–Meier curve according to HELLS. **(E)** Kaplan–Meier curve according to IL1B. **(F)** Kaplan–Meier curve according to MUC1. **(G)** Kaplan–Meier curve according to NNMT. **(H)** Kaplan–Meier curve according to TP63. **(I)** Kaplan–Meier curve according to PLIN2. **(J)** Kaplan–Meier curve according to TFR2. **(K)** Kaplan–Meier curve according to SMAD7. **(L)** Kaplan–Meier curve according to DPP4.

Excluding samples with incomplete clinical data and OS of less than 30 days, 482 patients from the TCGA were analyzed. As shown in **Figures 4A–L**, 12 genes associated with ferroptosis were ruled out as prognostic factors.

### Establishment and Validation of a Ferroptosis-Related Prognostic Signature

**Figure 5:**
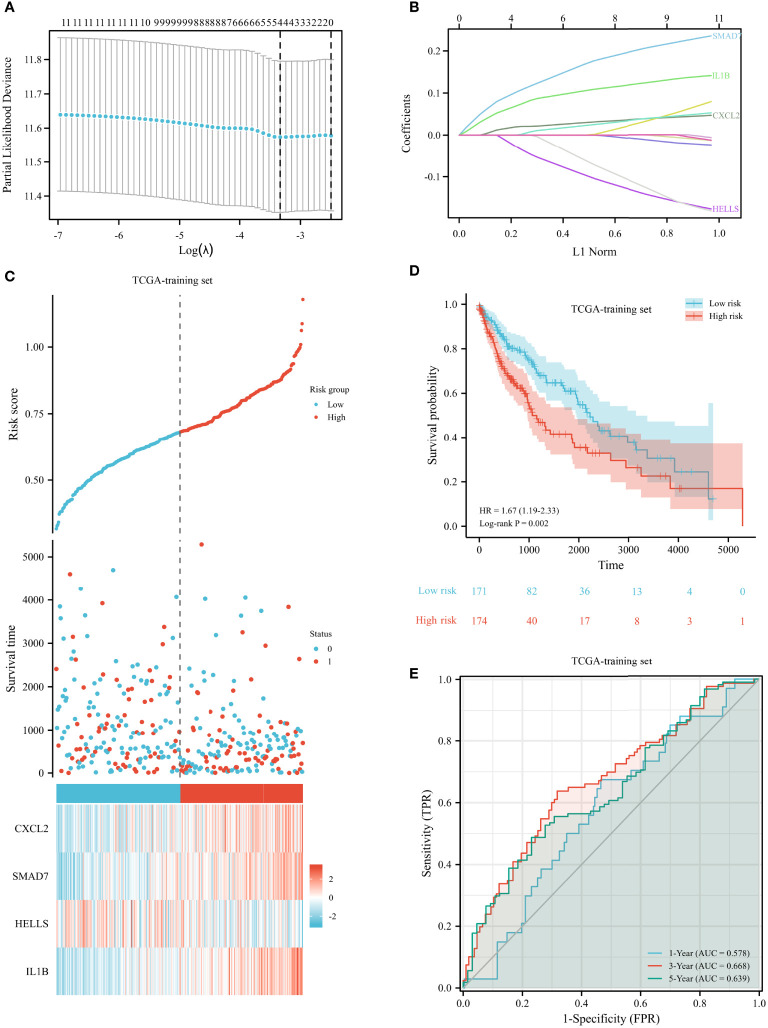
Construction of prognostic signature for LUSC in the TCGA training set. **(A)** The selection of optimal predictive variables. **(B)** LASSO coefficients of the 12 variables. **(C, D)** Risk plot **(C)** and overall survival **(D)** between the high-risk and low-risk groups. **(E)** The receiver operating curve for overall survival over time.

We randomly divided 482 LUSC patients from the TCGA database into a training set and a testing set at a 7:3 ratio. To validate our prognostic signature based on FRGs, we used GSE73403 as a validation cohort. A LASSO model was built from the TCGA training set to develop a prognostic model. The above 12 FRGs were further narrowed down to 4 genes, namely, CXCL2, SMAD7, HELLs, and IL1B (**Figures 5A, B**). The risk score was calculated as follows: 0.01967 × CXCL2 + 0.1017 × SMAD7 − 0.0274 × HELLS + 0.069 × IL1B. The risk scores of all patients were calculated. In accordance with the median risk score, patients were separated into two groups. The high-risk patients experienced a worse OS than low-risk patients (shown in **Figures 5C, D**). We created 3-year and 5-year ROC curves to determine the prognostic efficacy. The area under the curve (AUC) was 0.668 for 3 years and 0.646 for 5 years, suggesting that the model was well established (shown in **Figure 5E**).

**Figure 6:**
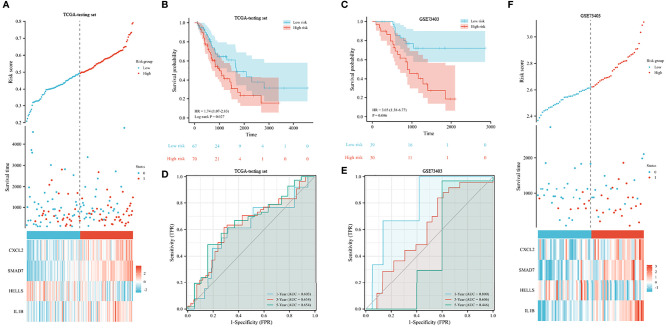
Validation of prognostic signature for LUSC. **(A, B)** Risk plot **(A)** and overall survival **(B)** in the TCGA testing set. **(C)** Overall survival analysis in the GEO validation set. **(D, E)** The receiver operating curve for overall survival in the TCGA testing set **(D)** and GEO validation set **(E)**. **(F)** Risk plot in the GEO validation set.

In both testing and validation sets, the proposed 4-gene prognostic model was further validated. The Kaplan–Meier survival plots of the test set indicated that the prognostic model could classify LUSC patients into high-risk and low-risk groups (**Figures 6A, B**). The patients with high-risk scores had significantly shorter survival times than low-risk patients (HR 1.74, 95% CI 1.07–2.83, *p* = 0.027). According to the results from the training and testing cohorts, high-risk LUSC patients in the external validation set had a worse OS (shown in **Figure 6C**). The AUC values were 0.603, 0.634, and 0.654 for 1, 3, and 5 years in the testing set (**Figure 6D**). In the external validation set, the 1-, 3-, and 5-year AUC values were 0.800, 0.606, and 0.446, respectively (**Figure 6E**). The risk plot of external dataset was shown in (**Figure 6F**), revealing the prognostic model could distinguish LUSC patients with high-risk well.

**Figure 7:**
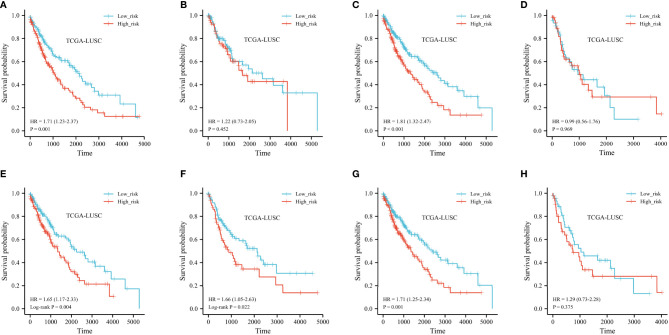
Kaplan–Meier curve of stratified analyses of the FRG prognostic signature for associations with clinical characteristics. **(A)** OS curve in patients older than 65 years old. **(B)** OS curve in patients younger than 65 years old. **(C)** OS curve in the T1+T2 stage. **(D)** OS curve in the T1+T2 stage. **(E)** OS curve in the N0 stage. **(F)** OS curve in the N+ stage. **(G)** OS curve in stage I + stage II. **(H)** OS curve in stage III + stage IV.

We performed stratified analyses of the FRG prognostic signature for associations with clinical parameters, including age, sex, and TNM stage. The Kaplan–Meier plot indicated that the prognostic risk model accurately classified LUSC patients into short-term and long-term survival groups among patients older than 65 years (shown in **Figure 7A**). However, among people younger than 65 years old, the HR for high-risk group patients was 1.22 (95% CI 0.73–2.05) (*p* = 0.452, shown in **Figure 7B**). When stratified by sex and N stage, the risk score was significantly associated with survival in both groups (shown in **Figures 7C–F**). The relationship was not noteworthy among people with late T stage disease. According to the TNM stage, no association between risk scores and survival was observed in LUSC patients with advanced stage, but high-risk patients with early stage had a worse prognosis (shown in **Figures 7G, H**). The forest plot of the univariate Cox regression is shown in **Supplementary Figure 1**.

### Ferroptosis-Related Gene Signature Related to Immune Cell Infiltration

**Figure 8:**
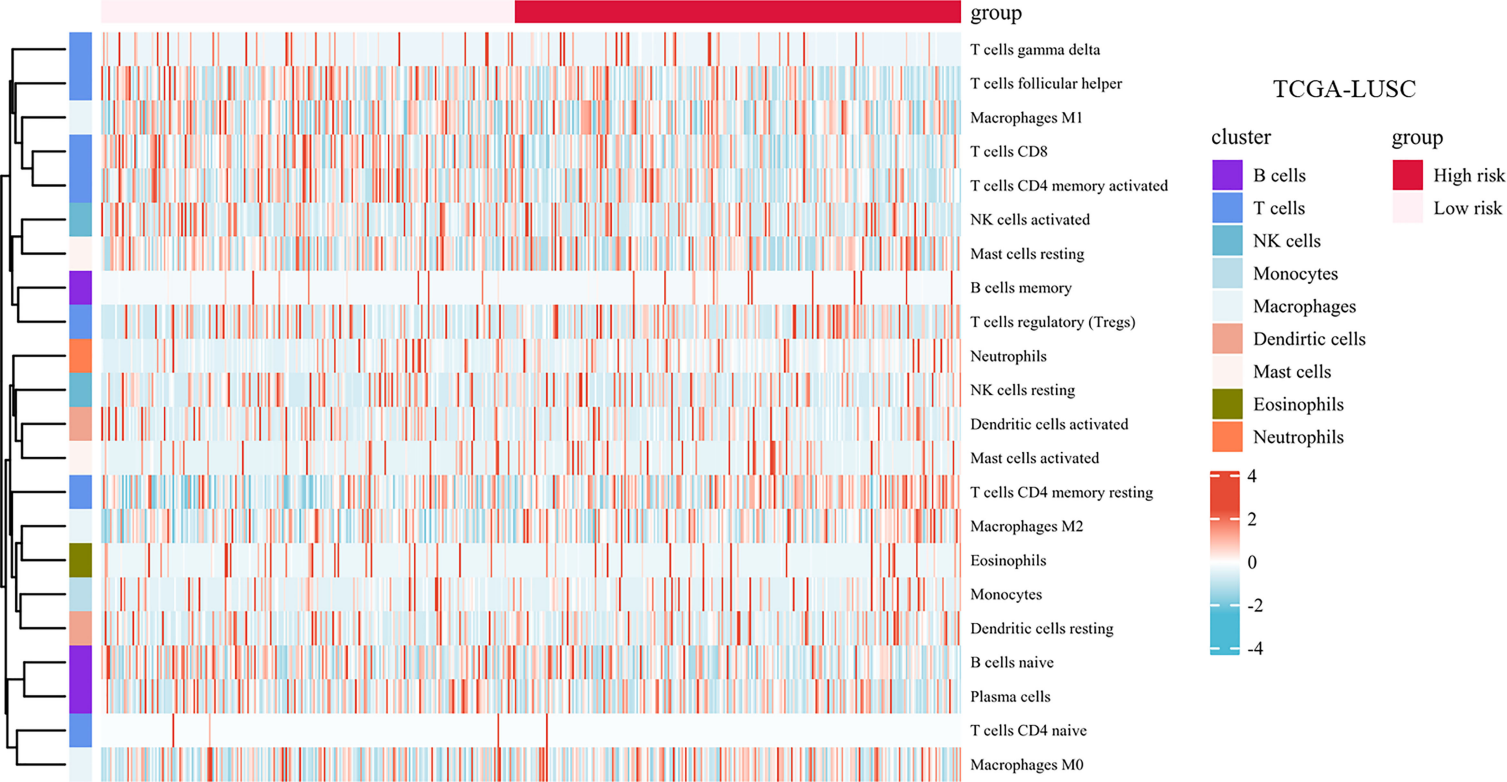
Infiltration of immune cells among high-risk groups versus low-risk groups.

**Figure 9:**
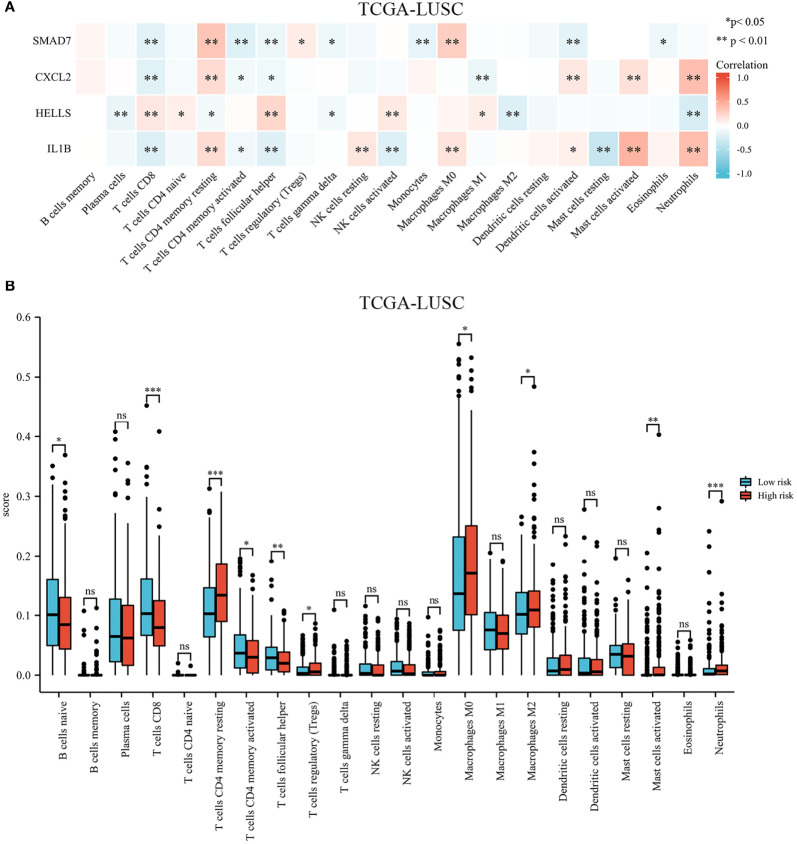
Immune cell infiltration in different high- and low-risk groups in the TCGA cohort. **(A)** Heatmap of immune cell proportions. **(B)** The bar graph showing the difference between infiltrated immune cells in the tumor microenvironment. *p < .05, **p < .01, ***p < .001, and ns means not significant.

The landscape of immune cell infiltration in the different groups in the TCGA cohort is demonstrated in a heatmap (**Figure 8**). There were significant differences in the SMAD7, CXCL2, HELLS, and IL1B expression levels among the infiltrating immune cells, including CD8^+^ T cells, follicular helper T cells, regulatory T cells, macrophages, and so on (shown in **Figure 9A**).

We compared the tumor-infiltrating immune cells between the low-risk and high-risk groups. The CIBERSORT was applied to evaluate the proportion of immune cells. The proportions of CD8^+^ T cells, CD4^+^ memory activated T cells, and follicular helper T cells were significantly increased in the low-risk LUSC patients (**Figure 9B**). Additionally, we observed that the relative fractions of regulatory T cells, M0 macrophages, M2 macrophages, and neutrophils were significantly increased in the high-risk group. However, there were no significant differences in gamma delta T cells, resting NK cells, activated NK cells, or dendritic cell infiltration between the two groups.

**Figure 10:**
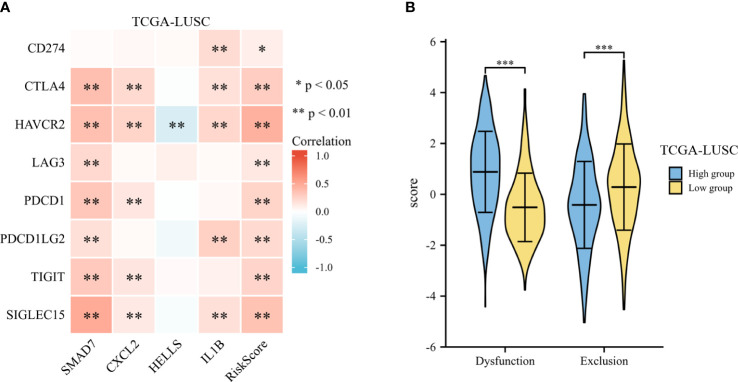
Immunotherapy response of LUSC. **(A)** The heatmap of expression of risk score and selected immune-checkpoint-relevant genes. **(B)** The violin plot for the TIDE scores between the high- and low-risk groups. *p < .05, **p < .01, and ***p < .001.

The expression of risk score and selected immune-checkpoint-relevant genes was illustrated in a heatmap (shown in **Figure 10A**). The risk score had a notable direct correlation with the expression of all these immune-checkpoint-relevant transcripts, indicating that the risk score represented the state of tumor-induced immunosuppression.

To predict the clinical outcome of immune checkpoint inhibitors, TIDE was used. TIDE scores differed significantly between high-risk and low-risk groups (shown in **Figure 10B**). According to TIDE, low-risk patients had a higher exclusion score and a lower dysfunction score than high-risk patients.

**Figure 11:**
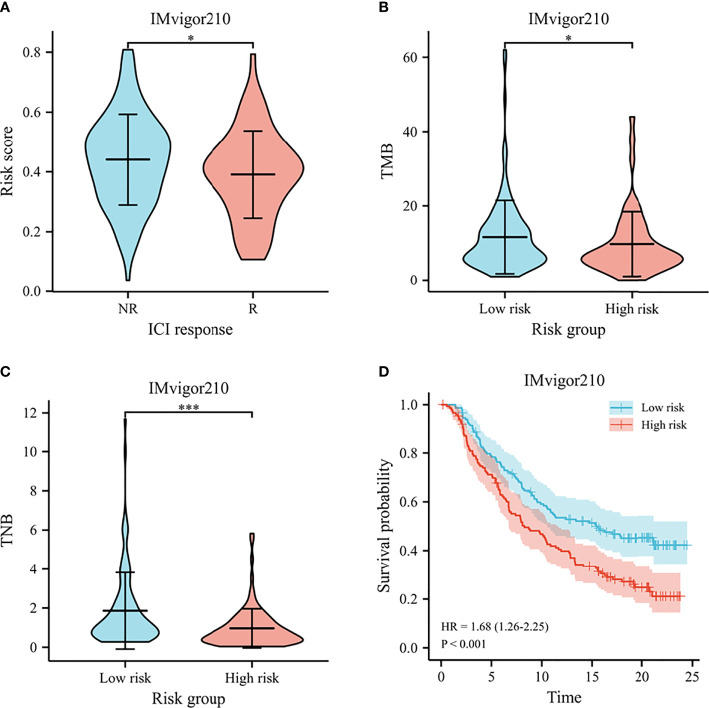
Immunotherapy response in IMvigor210. **(A)** The violin plot for risk scores between responders and non-responders to immunotherapy. **(B)** The violin plot for TMB between high- and low-risk groups. **(C)** The violin plot for TNB between high- and low-risk groups. **(D)** Survival curves of predicting the OS using risk score. *p < .05, and *** p < .001.

The IMvigor210 cohort investigated the molecular biomarkers that could predict the immunotherapy efficacy among muscle invasive bladder cancer patients. We further detected the prognostic signature in predicting immunotherapy response, including survival, therapy response, and immune checkpoints, in the IMvigor210 cohort. The risk scores were significantly different between non-responders and responders to immunotherapy (**Figure 11A**). Meanwhile, the low-risk patients harbored higher TMB and TNB than high-risk patients (**Figures 11B, C**). According to the Kaplan–Meier survival plots, patients could be classified into low- and high-risk groups based on the prognostic model (shown in **Figure 11D**).

### Construction of the Nomogram

**Figure 12:**
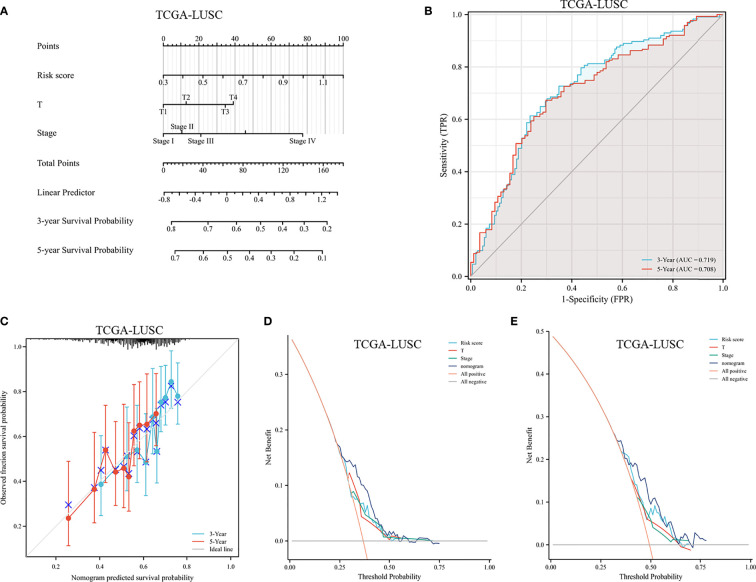
Assessment of a nomogram based on clinical characteristics and the risk scores. **(A)** A nomogram based on the risk model and clinical parameters. **(B)** Time-dependent receiver operating curve for predicting overall survival. **(C)** Calibration curve of the nomogram in the TCGA dataset at 3-year survival and 5-year survival. **(D, E)** DCA evaluating the clinical usefulness in the TCGA dataset at 3-year survival **(D)** and 5-year survival **(E)**.

A prognostic nomogram was established by integrating the FRG signature and clinical characteristics. According to the univariate Cox regression analyses, the risk score, sex, age, T stage, M stage, and clinical stage were potential independent prognostic factors (shown in **Supplementary Table 2**). In order to predict the survival rates over a 3- and 5-year period, we incorporated all of these factors into a nomogram. Based on the nomogram, the survival rate was assessed by summing several variables, including risk score, T stage, and clinical stage (**Figure 12A**). According to the nomogram, the AUCs over 3 and 5 years were 0.719 and 0.708, respectively (**Figure 12B**). Calibration curves of the nomogram indicated an accordant agreement for the 3-year and 5-year OS (**Figure 12C**). The DCA showed that if the threshold probability of a patient was >30%, using the nomogram to predict 3-year OS and 5-year OS demonstrated a larger benefit than did the clinical factors (**Figures 12D, E**). Overall, the prognostic nomogram was superior in predicting the survival outcomes of LUSC patients.

## Discussion

In recent decades, outcome predictions for LUSC patients have primarily relied on clinical characteristics, including age, TNM stage, and some serum tumor biomarkers. However, the predictive efficacy of these factors is limited and they are not able to assist in clinical decision-making. Therefore, the identification of more effective biomarkers could help physicians judge the prognosis and make subsequent individual treatment decisions. With advances in sequencing technology, genomics might be useful to identify predictive biomarkers in malignancies. However, one single gene offers little predictive power for the outcomes of LUSC patients. A number of multigene models have demonstrated much better predictive power than single genes.

Ferroptosis is a recently identified method of regulating cell death involving iron-dependent ROS generation (8). Recent studies have demonstrated that ferroptosis contributes to the progression of various cancers, including adrenocortical carcinoma, pancreatic carcinoma, renal cell carcinoma, and hepatocellular carcinoma (29, 30). According to Wang (31), immunotherapy-activated CD8+ T cells aggravated ferroptosis-specific lipid peroxidation, thereby enhancing immunotherapy’s anti-tumor efficacy. Zhang (15) showed that hydroxyl radicals caused lethal ferroptosis in tumor cells, and the immunogenicity of the microenvironment was enhanced by the exposed tumor antigens as a result. Ferroptosis is likely to play a role in tumor immunity. A previous study (32) showed that the ferroptosis regulators served as a prognostic factor for recurrence and survival in pan-cancer tissue. Furthermore, the ferroptosis score was an independent predictor of response to immunotherapy. Collectively, understanding mechanisms of ferroptosis in the tumor immune microenvironment in LUSC may facilitate biomarker-guided clinical decisions.

Using FRG signatures, we identified high-risk LUSC patients and investigated the relationship between the risk signature and the tumor immune microenvironment. First, we screened FRGs from the FerrDb database and identified 132 DEGs in the TCGA cohort. To investigate the molecular mechanisms and biological functions of these genes, we conducted functional analyses. In the TCGA cohort, twelve FRGs were screened out as prognostic genes by univariate Cox regression analysis. We then constructed a prognostic model of 4 FRGs *via* LASSO regression. A favorable predictive efficacy was demonstrated in both the TCGA testing set and the external validation set. Finally, we combined the clinical characteristics with a LASSO regression model to construct a nomogram that could be easily applied in clinical practice.

All four genes have been validated as ferroptosis-related. It has recently been proposed that ferroptosis is likely to release several immune modulators that trigger an anti-tumor immune response. Among the 4 FRGs in the prognostic model, CXCL2 is the ligand of the chemokine receptor CXCR2 and is mainly expressed on macrophages and myeloid-derived neutrophils. CXCL2 can recruit myeloid-derived suppressor cells and tumor-associated macrophages and increase immunosuppressive effects, thus enhancing cancer cell proliferation, invasion, and metastasis (33, 34). CXCL2 has been reported to facilitate myeloid cell migration and CD8^+^ T-cell exhaustion, causing accelerated tumor growth and invasion in glioblastoma (35). In the context of hepatocellular carcinoma, CXCL2 is downregulated in tumor tissues compared to adjacent normal tissues, and upregulation of CXCL2 inhibited angiogenesis and the aggressiveness of hepatocellular carcinoma (36). However, Peng (37) found that CXCL2 was a major chemokine involved in regulating the recruitment of neutrophils into the tumor immune microenvironment and promoting the production of prometastatic factors with positive feedback. At present, there is a relative dearth of studies exploring the role of CXCL2 in the tumor immune microenvironment of LUSC.

Previous studies have revealed that SMAD7 acts as a tumor suppressor in a variety of cancers, including gastric cancer (38), bladder cancer (39), and hepatocellular carcinoma (40). Nevertheless, several studies have pointed out that SMAD7 might promote tumor progression, migration, and invasion. SMAD7 enhanced TGF-β induction of c-Jun and HDAC6 and contributed to tumor aggressiveness in prostate cancer cells (41). In colorectal cancer, SMAD7 expression was associated with poorly differentiated cell morphology, higher cell proliferation, and liver metastases (42). Luo (43) found that overexpression of SMAD7 overexpression increased lung cancer incidence. Zhou (44) reported that overexpression of SMAD7 promoted proliferative and migratory capacities in pancreatic cancer. Taken together, the literature on the involvement of SMAD7 in tumor progression has reported contradictory data about its protumorigenic or antitumorigenic role in different types of cancer. In our study, the expression of SMAD7 was negatively associated with CD8^+^ T cells and positively associated with regulatory T cells and M0 macrophages. Those with high SMAD7 expression have significantly shorter OS compared to those with low expression. Taken together, we speculated that SMAD7 overexpression might contribute to immunological suppression and a poor prognosis in LUSC. Additional studies are required to clarify the exact mechanism of SMAD7.

HELLS, a member of the SNF2 chromatin remodeling protein family, modifies the nucleosome organization and position by disrupting histone–DNA interactions (45). HELLS has been reported to maintain cancer cell stemness by controlling DNA methylation patterns (46). Overexpression of HELLS enables continuous cell cycle activity and proliferation and is associated with poor outcomes. Hou (47) revealed that HELLS might serve as an oncogene in pancreatic cancer, and downregulating HELLS impaired tumor growth. In hepatocellular carcinoma, HELLS is involved in chromatin remodeling and epigenetic silencing, thus promoting tumor proliferation and metastasis (48). Additionally, Zhu (49) found that high expression of HELLS was related to an improved prognosis in lung cancer patients. Xing (50) reported that HELLS expression levels were correlated with the OS of cervical carcinoma and endocervical adenocarcinoma. Our results suggested that patients with high HELLS expression displayed better OS. In conclusion, these results indicated that HLELS plays different roles in different types of cancers.

IL-1β, a member of the IL-1 cytokine family, plays a critical role in cytokine production, cellular migration, angiogenesis, and the immune response. In pancreatic cancer, IL-1β activated quiescent pancreatic stellate cells and promoted an immunosuppressive microenvironment rich in M2 macrophages, myeloid-derived suppressor cells, regulatory B cells, and Th17 cells (51). Kaplanov (52) found that implanted breast cancer tumors regressed in IL-1β-deficient mice or in wild-type mice treated with anti-IL-1β antibodies. Furthermore, the blockade of IL-1β and PD-1 completely abrogated the tumors.

The regulatory T cells were related with worse prognosis in various solid tumors, including breast cancer, pancreatic cancer, and ovarian cancer (53). Tian (54) reported that the regulatory T cells were correlated with histopathological grade in gliomas, indicating that the regulatory T cells might play a crucial role in carcinogenesis. Cui (55) found that follicular helper T cells were critical for germinal center formation by supporting B cells and correlated with prolonged survival in lung adenocarcinoma patients. However, Eschweiler (56) found that follicular helper T cells were prevalent in multiple tumors, including NSCLC, melanoma, breast cancer, and colorectal cancer. They were located in tertiary lymphoid structures and presented superior immunosuppressive capacity. A previous study revealed that M0 and M2 macrophages were independent prognostic factors and associated with a high risk of relapse in multiple solid tumors, including colorectal cancer (57), breast cancer (58), and glioblastoma (59). Based on our findings, the proportions of anti-tumor immune cells, including CD8^+^ T cells, CD4^+^ memory activated T cells, and follicular helper T cells, were significantly increased in the low-risk group compared with the high-risk group. Meanwhile, the immunosuppressive cells, including regulatory T cells, M0 macrophages, and M2 macrophages, were significantly accumulated in the high-risk group.

The risk score exhibited a significantly positive relation with all these immune-checkpoint-relevant transcripts, indicating that the risk score represented the state of tumor-induced immunosuppression. We further estimated the TIDE algorithm to identify patients who might benefit from ICIs. We found that high-risk LUSC patients with a higher dysfunction score and a lower exclusion score might benefit less from ICIs than low-risk LUSC patients. The results of immune-checkpoint-relevant transcripts and TIDE algorithm were consistent with CIBERSORT, indicating that the high-risk LUSC patients more likely exhibited an immunosuppressive tumor microenvironment.

The study has important implications for the prognosis and treatment for LUSC patients. Above all, we provided a new FRG signature to guide clinical practice and risk stratification. The patients with a low risk score were more likely to benefit from ICIs and experienced longer survival time. Secondly, we identified several critical ferroptosis genes that might offer therapeutic targets in LUSC. The previous study (60) searched for FRGs in LUSC patients. They identified 16 genes and built a risk model for OS. Instead, we constructed a 4-gene prognostic model with the desired efficacy. Feng (61) constructed an algorithm based on FRGs and explored the relation of ferroptosis score and immunotherapy response among LUSC patients. Aside from research on immunotherapy response and prognosis among LUSC patients, the present study further explored the prognostic value of the model among other solid tumors.

Our study has some limitations. First, an open-source database was used to download mRNA expression data and related clinical information. These findings have yet to be verified in clinical trials. Additionally, lung squamous cancer is a complex disease regulated by multiple factors, such as the environment, genetics, and epigenetics. Additional molecular biological experiments are required to confirm that the 4 FRGs have roles in the progression of LUSC. Finally, the risk model could not provide the prognostic value compared to several commonly used predictors, including pathological grade and treatment strategy, because of missing TCGA data.

In summary, we identified an FRG signature to predict the OS of LUSC patients. This risk signature has utility as a clinical prognostic tool for guiding clinical practice and risk stratification. Furthermore, the low- and high-risk groups identified by the signature had different degrees of immune cell infiltration and response to immunotherapy. Thus, this prognostic model provides an accurate and objective basis for guiding individualized treatment decisions for LUSC.

## Data Availability Statement

The original contributions presented in the study are included in the article/**Supplementary Material**. Further inquiries can be directed to the corresponding author.

## Author Contributions

QW and YC designed the study. BZ, HF, and WG collected the data. HW and HL performed the research. QW wrote the paper. YC, MX, and YD analyzed the data. All authors contributed to the article and approved the submitted version.

## Funding

This work was supported by the Nature Science Foundation of Shandong Province (ZR2018BH019) and the National Natural Science Foundation of China (82003224). The funder had no role in study design, data collection and analysis, decision to publish, or preparation of the manuscript.

## Conflict of Interest

The authors declare that the research was conducted in the absence of any commercial or financial relationships that could be construed as a potential conflict of interest.

## Publisher’s Note

All claims expressed in this article are solely those of the authors and do not necessarily represent those of their affiliated organizations, or those of the publisher, the editors and the reviewers. Any product that may be evaluated in this article, or claim that may be made by its manufacturer, is not guaranteed or endorsed by the publisher.

## Supplementary Material

Supplementary Figure 1 The forest plot of univariate Cox regression.
