# Supplementary figures and images for: Identification and Validation of a Four-Gene Ferroptosis Signature for Predicting Overall Survival of Lung Squamous Cell Carcinoma
Source: Front Oncol. 2022 Jul 7;12:933925. doi: 10.3389/fonc.2022.933925 (PMC9330609; doi:10.3389/fonc.2022.933925)

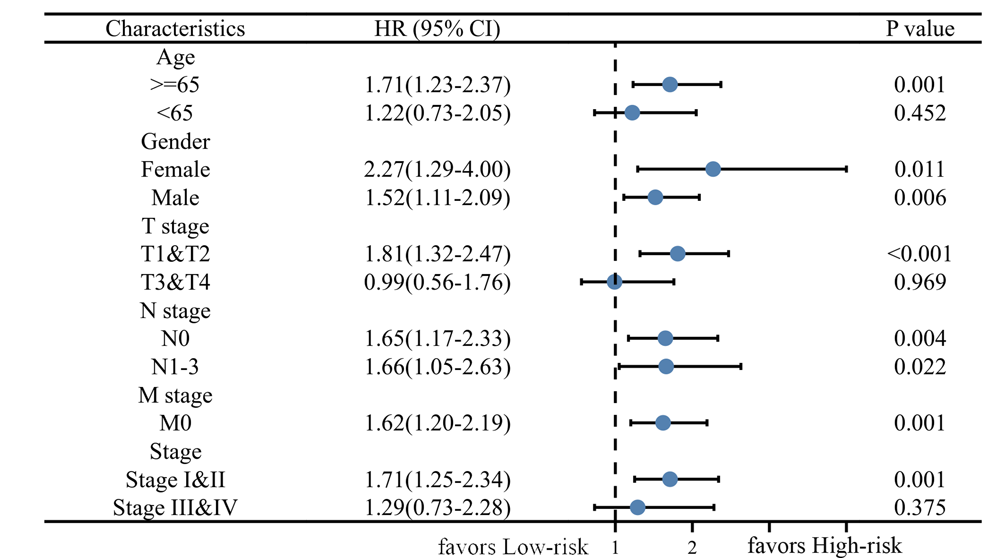

Supplement: Supplementary Figure 1 — The forest plot of univariate Cox regression. [file Image_1.tif]
