# Supplementary material for: Identification and Validation of a Four-Gene Ferroptosis Signature for Predicting Overall Survival of Lung Squamous Cell Carcinoma
Source: Front Oncol. 2022 Jul 7;12:933925. doi: 10.3389/fonc.2022.933925 (PMC9330609; doi:10.3389/fonc.2022.933925)
Supplement: Supplementary file 2 [file Table_1.docx]

Supplementary Table1. Ferroptosis-related genes.

| PTGS2 | SCP2 | CGAS | MAPK14 |
| --- | --- | --- | --- |
| DUSP1 | TP53 | STING1 | LINC00472 |
| NOS2 | ACSL4 | HDDC3 | NOX4 |
| NCF2 | LPCAT3 | MIR761 | GOT1 |
| MT3 | NRAS | MDM2 | BECN1 |
| UBC | KRAS | MDM4 | PRKAA2 |
| ALB | HRAS | ALOX15 | PRKAA1 |
| TXNRD1 | TF | POR | ELAVL1 |
| SRXN1 | TFRC | MIR214 | BAP1 |
| GPX2 | TFR2 | DLD | TP53 |
| BNIP3 | SLC38A1 | LONP1 | ABCC1 |
| OXSR1 | SLC1A5 | BACH1 | ACSL4 |
| SELENOS | GLS2 | DNAJB6 | MIR6852 |
| ANGPTL7 | GOT1 | WWTR1 | ACVR1B |
| CHAC1 | CARS1 | ATM | TGFBR1 |
| SLC7A11 | TP53 | PRKCA | BAP1 |
| DDIT4 | ALOX5 | BECN1 | EPAS1 |
| LOC284561 | KEAP1 | FBXW7 | HILPDA |
| ASNS | HMOX1 | PANX1 | HIF1A |
| TSC22D3 | TP53 | DNAJB6 | ALOX12 |
| DDIT3 | TP53 | BACH1 | ACSL4 |
| JDP2 | GLS2 | ACSL4 | HMOX1 |
| SESN2 | ATG5 | LONP1 | IFNG |
| SLC1A4 | ATG7 | CD82 | ANO6 |
| PCK2 | NCOA4 | IL1B | LPIN1 |
| TXNIP | TF | CTSB | HMGB1 |
| VLDLR | ALOX5 | POR | TNFAIP3 |
| GPT2 | ALOX12 | CYB5R1 | TLR4 |
| PSAT1 | ALOX12B | ELOVL5 | NOX4 |
| LURAP1L | ALOX15 | FADS1 | ATF3 |
| SLC7A5 | ALOX15B | ALOX12 | ATM |
| HERPUD1 | ALOXE3 | FBW7 | YY1AP1 |
| XBP1 | PHKG2 | PTEN | EGLN2 |
| ATF3 | TFRC | NR1D1 | MIOX |
| SLC3A2 | ACO1 | NR1D2 | TAZ |
| CBS | IREB2 | TBK1 | MTDH |
| ATF4 | SLC38A1 | IL6 | IDH1 |
| ZNF419 | GLS2 | USP7 | SIRT1 |
| KLHL24 | G6PDX | miR-182-5p | TAZ |
| TRIB3 | ULK1 | miR-378a-3p | ATF4 |
| ZFP69B | ATG3 | CTSB | BECN1 |
| ATP6V1G2 | ATG4D | ACSL4 | AQP3 |
| VEGFA | AQP8 | LINC00618 | AQP5 |
| GDF15 | RB1 | GLUT13 | AIFM2 |
| TUBE1 | HSPB1 | SLC2A14 | AIFM2 |
| ARRDC3 | HSF1 | EIF2AK4 | LAMP2 |
| CEBPG | SLC7A11 | EIF2S1 | ZFP36 |
| SNORA16A | GPX4 | ATF4 | GPX4 |
| RGS4 | GCLC | ALOX5 | PROM2 |
| BLOC1S5-TXNDC5 | SLC7A11 | ALOX12 | CHMP5 |
| LOC390705 | NFE2L2 | ALOX15 | CHMP6 |
| EIF2S1 | SQSTM1 | ALOX5 | AKR1C1 |
| KIM-1 | NQO1 | ACSF2 | AKR1C2 |
| IL6 | HMOX1 | IREB2 | AKR1C3 |
| CXCL2 | FTH1 | GPX4 | CBS |
| RELA | MUC1 | HMGB1 | NFE2L2 |
| HSD17B11 | SLC3A2 | HMOX1 | CAV1 |
| AGPAT3 | MT1G | NFE2L2 | GCH1 |
| SETD1B | NFE2L2 | ELAVL1 | SIRT3 |
| HMOX1 | SLC40A1 | SLC3A2 | DAZAP1 |
| TF | SLC7A11 | SLC7A11 | PIR |
| FTL | GPX4 | TFAP2C | GCLC |
| RPL8 | SLC7A11 | SP1 | FTL |
| ATP5MC3 | CISD1 | HBA1 | HCAR1 |
| TFRC | SLC7A11 | NNMT | SLC16A1 |
| MAFG | FANCD2 | PLIN4 | RRM2 |
| IL33 | GPX4 | HIC1 | SCD |
| FTH1 | NFE2L2 | STMN1 | NR4A1 |
| SLC40A1 | FTMT | RRM2 | PIK3CA |
| TF | HSPA5 | CAPG | RPTOR |
| TFRC | ATF4 | HNF4A | SREBF1 |
| FTH1 | SLC7A11 | NGB | SREBF2 |
| GPX4 | GPX4 | YWHAE | FZD7 |
| HAMP | GPX4 | GABPB1 | NFE2L2 |
| HSPB1 | HMOX1 | AURKA | NFE2L2 |
| NFE2L2 | ATF4 | MIR4715 | P4HB |
| STEAP3 | NFE2L2 | RIPK1 | NT5DC2 |
| DRD5 | TP53 | PRDX1 | BCAT2 |
| GPX4 | SLC7A11 | MIR30B | HSF1 |
| DRD4 | HELLS | MMP13 | PLA2G6 |
| MAP3K5 | SCD | LRRFIP1 | MIR424 |
| MAPK14 | FADS2 | SLC7A11 | PARK7 |
| SLC2A1 | SRC | GPX4 | FXN |
| SLC2A3 | STAT3 | AKR1C1 | SUV39H1 |
| SLC2A6 | NFE2L2 | AKR1C2 | ATF2 |
| SLC2A8 | PML | AKR1C3 | CDKN1A |
| SLC2A12 | MTOR | GPX4 | FTH1 |
| NFS1 | TFAM | NFE2L2 | ATG5 |
| TP63 | KDM3B | STAT3 | BECN1 |
| SLC7A11 | RNF113A | ACOT1 | MAP1LC3A |
| TP53 | PARK7 | NFE2L2 | GABARAPL2 |
| CDKN1A | AHCY | ALDH3A2 | GABARAPL1 |
| MIR137 | FXN | NFE2L2 | ATG16L1 |
| SLC40A1 | circ-TTBK2 | STK11 | WIPI1 |
| GPX4 | MIR522 | FNDC5 | WIPI2 |
| GPX4 | IDH2 | CircIL4R | SNX4 |
| ENPP2 | PPARA | CDH1 | ATG13 |
| VDAC2 | NOS2 | NFE2L2 | ULK2 |
| FH | SIAH2 | MIR214 | NCOA4 |
| CISD2 | RELA | NEDD4L | ACSL4 |
| SLC40A1 | PRKAA2 | SQSTM1 | TP53 |
| MIR9-1 | VDR | TF | SAT1 |
| MIR9-2 | NEDD4 | FTMT | ALOX15 |
| MIR9-3 | FXN | BRD2 | ACSL4 |
| CBS | AIFM2 | PIK3CA | LPCAT3 |
| NFE2L2 | PRDX1 | BRD3 | ALOX15 |
| SQSTM1 | AR | BRD4 | ACSL4 |
| GPX4 | CBS | BRDT | KEAP1 |
| ISCU | NFE2L2 | SCD | EGFR |
| FTH1 | CHMP5 | SLC7A11 | NOX4 |
| ACSL3 | CHMP6 | DECR1 | MAPK3 |
| OTUB1 | HMOX1 | NFE2L2 | MAPK1 |
| CD44 | ZFP36 | GPX4 | BID |
| LINC00336 | LAMP2 | SLC7A11 | ACSL4 |
| STAT3 | MTF1 | NFE2L2 | ZEB1 |
| BRD4 | RPL8 | GLRX5 | KEAP1 |
| PRDX6 | IREB2 | GPX4 | DPP4 |
| MIR17 | ATP5MC3 | NCOA3 | ALOX15 |
| SCD | CS | NR5A2 | ALOX12 |
| SESN2 | EMC2 | FLT3 | CDKN2A |
| NF2 | ACSF2 | GPX4 | PEBP1 |
| ARNTL | NOX1 | MTOR | SOCS1 |
| HIF1A | CYBB | PANX2 | CDO1 |
| JUN | NOX3 | RHEBP1 | MYB |
| CA9 | NOX4 | TFAP2A | HMOX1 |
| HSPA5 | NOX5 | CP | MAPK8 |
| TMBIM4 | DUOX1 | SLC7A11 | MAPK9 |
| HSPA5 | DUOX2 | ARF6 | MAPK1 |
| PLIN2 | G6PD | GDF15 | MAPK3 |
| MIR212 | PGD | ABHD12 | SLC1A5 |
| Fer1HCH | VDAC2 | PPP1R13L | CHAC1 |
| IREB2 | GSK3B |  |  |
| MT1DP | MAPK8 |  |  |
| ACSL4 | BRD7 |  |  |
| PEX10 | TP53 |  |  |
| KEAP1 | SLC25A28 |  |  |
| AGPAT3 | ACSL4 |  |  |
| PEX12 | MFN2 |  |  |
| CHP1 | SLC11A2 |  |  |
| GPAT4 | ZFAS1 |  |  |
| BRPF1 | SLC38A1 |  |  |
| OSBPL9 | TSC1 |  |  |
| INTS2 | PEBP1 |  |  |
| MMD | TGFB1 |  |  |
| CYP4F8 | SNCA |  |  |
| MLLT1 | SIRT3 |  |  |
| TTPA | PRKAA2 |  |  |
| GRIA3 | TFRC |  |  |
| EPT1 |  |  |  |
| POM121L12 |  |  |  |
| LIG3 |  |  |  |
| AEBP2 |  |  |  |
| AGPS |  |  |  |
| CDCA3 |  |  |  |
| PEX2 |  |  |  |
| LPCAT3 |  |  |  |
| PEX6 |  |  |  |
| TIMM9 |  |  |  |
| DCAF7 |  |  |  |
| LCE2C |  |  |  |
| FAR1 |  |  |  |
| PHF21A |  |  |  |
| SMAD7 |  |  |  |
| LYRM1 |  |  |  |
| AMN |  |  |  |
| PEX3 |  |  |  |
| MTCH1 |  |  |  |
| ZEB1 |  |  |  |
| SIRT1 |  |  |  |
| ACADSB |  |  |  |
| PVT1 |  |  |  |
| hsa_circ_0008367 |  |  |  |
| SLC39A14 |  |  |  |
| NCOA4 |  |  |  |
| MAP3K11 |  |  |  |
