# Supplementary material for: Identification and Validation of a Four-Gene Ferroptosis Signature for Predicting Overall Survival of Lung Squamous Cell Carcinoma
Source: Front Oncol. 2022 Jul 7;12:933925. doi: 10.3389/fonc.2022.933925 (PMC9330609; doi:10.3389/fonc.2022.933925)
Supplement: Supplementary file 3 [file Table_2.docx]

Supplementary Table 2. The univariate Cox regression analyses of the risk model and overall survival of LUSC patients in TCGA cohort

| Characteristics | Univariate analysis | |
| --- | --- | --- |
|  | Hazard ratio (95% CI) | P value |
| gender |  |  |
| MALE | Reference |  |
| FEMALE | 0.702 (0.477-1.033) | 0.073 |
| Age | 1.032 (1.011-1.054) | **0.003** |
| Number of pack-years smoked | 1.002 (0.997-1.007) | 0.399 |
| T |  |  |
| T1 | Reference |  |
| T2 | 1.044 (0.709-1.536) | 0.828 |
| T3 | 0.976 (0.541-1.760) | 0.935 |
| T4 | 1.967 (0.975-3.968) | 0.059 |
| N |  |  |
| N0 | Reference |  |
| N1 | 0.986 (0.684-1.422) | 0.940 |
| N2 | 1.159 (0.661-2.032) | 0.606 |
| N3 | 2.628 (0.642-10.752) | 0.179 |
| M |  |  |
| M0 | Reference |  |
| M1 | 2.659 (0.979-7.224) | 0.055 |
| Stage |  |  |
| StageI | Reference |  |
| StageII | 1.103 (0.761-1.600) | 0.604 |
| StageIII | 1.278 (0.836-1.955) | 0.257 |
| StageIV | 2.869 (1.041-7.903) | **0.041** |
| RiskScore | 3.482 (2.004-6.050) | **<0.001** |
